# Draft Genome Sequence of the Carbofuran-Mineralizing *Novosphingobium* sp. Strain KN65.2

**DOI:** 10.1128/genomeA.00764-15

**Published:** 2015-07-09

**Authors:** Thi Phi Oanh Nguyen, René De Mot, Dirk Springael

**Affiliations:** a Department of Earth and Environmental Sciences, Division of Soil and Water Management, Heverlee, Leuven, Belgium; b Centre of Microbial and Plant Genetics, Heverlee, Leuven, Belgium

## Abstract

Complete mineralization of the *N*-methylcarbamate insecticide carbofuran, including mineralization of the aromatic moiety, appears to be confined to sphingomonad isolates. Here, we report the first draft genome sequence of such a sphingomonad strain, i.e., *Novosphingobium* sp. KN65.2, isolated from carbofuran-exposed agricultural soil in Vietnam.

## GENOME ANNOUNCEMENT

Carbofuran (2,3-dihydro-2,2-dimethyl-7-benzofuranyl methyl carbamate) is a broad-spectrum systemic insecticide that has been used worldwide for disease control in vegetable, fruit, and forest crops. Due to its adverse effects on nontarget organisms, affecting the mammalian nervous (1, 2), reproductive (2), and excretory systems (3), carbofuran was recently banned in many countries, but the compound is still used in many developing countries. Various carbofuran-degrading bacteria have been isolated from carbofuran-treated soils, but most of them release only the *N*-methylated carbamate side chain of the compound and use this moiety for growth, resulting in an accumulation of carbofuran phenol (4, 5). Only sphingomonads have been documented to possess the capacity to degrade the aromatic ring structure of carbofuran (6, 7). In this paper, we report the genome sequence of the carbofuran-mineralizing strain *Novosphingobium* sp. KN65.2 (LMG 28221). Strain KN65.2 was isolated by enrichment in mineral medium, with carbofuran as the sole carbon source, from soil sampled at a vegetable field with a long history of carbofuran treatment and located in the Soc Trang province in the Mekong Delta of Vietnam (8).

The genome sequence of *Novosphingobium* sp. KN65.2 was determined by the Illumina GAIIx sequencing platform BaseClear (The Netherlands). The CLC bio Genomics Workbench (Qiagen) was used to assemble the 50-bp paired-end reads, yielding 243 contigs, with an average length of 20.6 kb and an average coverage of 58.7. The total assembled length is 5,024,847 bp, with a G+C content of 63.1%. The draft genome sequence of strain KN65.2 was annotated by MaGe (https://www.genoscope.cns.fr/agc/microscope/home/index.php), revealing 5,167 protein-coding sequences and 49 RNA genes (3 rRNAs and 46 tRNAs).

Within the *Novosphingobium* genus, strain KN65.2 phylogenetically clusters with *Novosphingobium pentaromativorans* US6-1 (9) and *Novosphingobium* sp. PP1Y (10), two related marine isolates that are capable of degrading polycyclic aromatic hydrocarbons (11, 12). *Novosphingobium* sp. KN65.2 possesses a large complement of oxygenase genes, mostly consisting of homologues found in other sphingomonads. Among the 17 annotated monooxygenase gene products are five members of the nitronate monooxygenase family that are possibly involved in nitroalkane metabolism and an alkanesulfonate monooxygenase (SsuD homologue) potentially mediating the catabolism of sulfonated compounds. An even larger number of putative dioxygenases (34) is encoded by the KN65.2 genome, including a member of the taurine catabolism dioxygenase family and a representative of the phytanoyl-coenzyme A (CoA) dioxygenase family that are common to many sphingomonads (12). More than half of the dioxygenases (19) belong to the glyoxalase/bleomycin/dioxygenase family. The CfdE enzyme involved in carbofuran catabolism represents a new member of this family. Equally unique to strain KN65.2 and contributing to carbofuran degradation is CfdI, belonging to the TfdB flavoprotein monooxygenase family (13). These enzymes enable the utilization of carbofuran as a carbon and nitrogen source by KN65.2 (8). *Novosphingobium* KN65.2 also carries an orthologue of *cehA*, encoding the carbaryl hydrolase of *Rhizobium* sp. strain AC100 (14).

### Nucleotide sequence accession numbers. 

This whole-genome project was deposited at the European Nucleotide Archive under accession no. CCBH000000000. The version reported in this paper is the first version, CCBH010000000.
